# scRNA-seq revealed the special TCR β & α V(D)J allelic inclusion rearrangement and the high proportion dual (or more) TCR-expressing cells

**DOI:** 10.1038/s41419-023-06004-7

**Published:** 2023-07-31

**Authors:** Lanwei Zhu, Qi Peng, Jun Li, Yingjie Wu, Jiayi Wang, Dewei Zhou, Long Ma, Xinsheng Yao

**Affiliations:** Department of Immunology, Center of Immunomolecular Engineering, Innovation & Practice Base for Graduate Students Education, Zunyi Medical University, Zunyi, China

**Keywords:** VDJ recombination, Immunogenetics

## Abstract

Allelic exclusion, one lymphocyte expresses one antigen receptor, is a fundamental mechanism of immunological self-tolerance and highly specific immune responses to pathogens. However, the phenomenon of V(D)J allelic inclusion (incomplete allelic exclusion or allelic escape) rearrangement and dual TCR T cells have been discovered by multiple laboratories. Despite continuous new discoveries, the proportion and underlying mechanism of dual TCR has been puzzling immunologists. In this study, we observed the presence of single T cells expressing multiple TCR chains in all samples, with the proportion of 15%, 10%, and 20% in the human thymus, human peripheral blood, and mouse lymphoid organs, respectively. The proportion of T cells possessing multiple T-cell receptors (TCR) varied significantly in different physiological states and developmental stages. By analyzing RSS category, RSS direction, and V(D)J gene position at TR locus of T cells which contain multiple TCR chains, we creatively found that one of TCR β (or TCR α) should originate from the transcription of V(D)J combination in T-cell receptor excision circle (TREC) formed after the twice successful rearrangement in the same chromosome. Moreover, human V30 (or mouse V31) gene may participate in reverse recombination and transcription to prevent allelic exclusion. In general, high proportion of T cells with multiple TCR at the transcriptome level was first made public, and we proposed a novel mechanism of secondary (or more) TCR rearrangement on a single chromosome. Our findings also indicated that the single-cell sequencing data should be classified according to the single, multiple, and abnormal TCR when analyzing the T-cell repertoire.

## Introduction

The clonal selection theory, which states that each lymphocyte expresses only one specific antigen receptor, serves as the foundation for T and B-cell development, tolerance selection, and specific immune responses. The theory primarily relies on the mechanism of allelic exclusion during TCR or BCR V(D)J gene rearrangement where only one allele assembles a functional gene. Consequently, mature T (or B) cells express a functional alpha (light) and beta (heavy) chain. T (or B) cells that fail to adhere to the rearrangement rules and self-tolerance selection undergo apoptosis [1]. The mechanism of allelic exclusion is regulated by various factors such as including feedback inhibition [2], pTα chain [3], protein kinase [4], cis-acting elements [5], transcription factor [6], and the quality of recombination signal sequence [7, 8] etc. Several hypothetical mechanisms, such as asynchronous recombination models, stochastic models, and feedback inhibition models [9], have been widely acknowledged. However, the precise mechanism of allelic exclusion remains incompletely understood [10], and the existence of single T (or B) cells expressing dual TCR (or BCR) challenges this theory.

Incomplete V(D)J allelic exclusion (or allelic inclusion, or allelic exclusion escape) was first found on B cells. In 1961, MAKELA et al. found that 4 of 455 cells can produce anti H and O antibodies at the same time [11]. Subsequently, B cells equipped with two functional V_H_DJ_H_ or V_K_J_K_/V_λ_J_λ_ were found in both human and mouse with different proportions (1–20%) [12–15]. Single-cell sequencing of human peripheral blood also observed two or more V_H_DJ_H_ (5.90–8.71%) or V_L_J_L_ (9.71–13.07%) recombination patterns of IgH chain or IgL chain, and each Ig class showed unique V_H_DJ_H_ recombination pattern in a single B-cell expressing multiple Ig classes [16]. In addition to physiological conditions, dual BCR B cells have been intensively studied in plasma cell tumors and other B-cell-related tumors [17–19]. Recently, the relationship of single lymphocytes expressing dual BCR chains or simultaneous expression of “TCR and BCR” in autogenetic immune diseases was emphasized: a quarter of SLE subjects showed higher frequency B cells with dual BCR [20], 2.2% lymphocytes possess dual TCR and BCR in type 1 diabetes [21].

Extensive experiments have provided support for the existence of single T cells expressing two functional α (V–J) and β chain (V-D-J). In 1988, two productive Vα and Vβ gene rearrangements in monoclonal T cells were detected at mRNA level [22, 23], which raised questions concerning the level at which allelic exclusion operates in T cells. Then, several laboratories have confirmed the incomplete V(D)J allelic exclusion of TCR chain and T-cell expresses more than one TCR both in human and mouse central and peripheral immune organs [22–28]. However, the proportion of T cells with dual TCR varies significantly (ranging from 0.01 to 30%) due to differences in research subjects and methods.

**Fig. 1:**
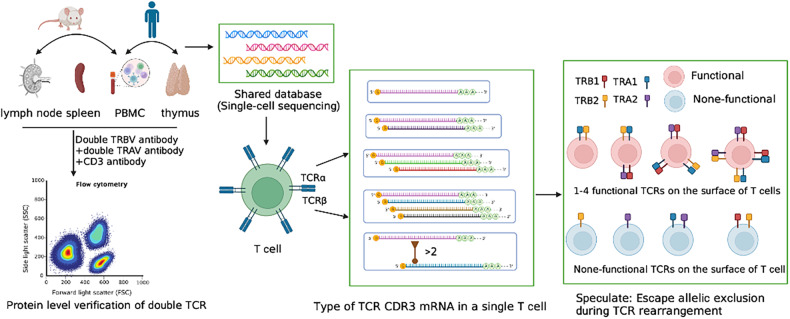
Illustration of experimental procedures.

Single-cell sequencing presents valuable opportunities for investigating the rearrangement of single T and B cells that possess more than two receptor chains. The characteristics and proportion of rearranged V(D)J can be analyzed from the transcriptome of an individual lymphocyte. In this study, we systematically analyzed the expression of TCR chains in 34 single-cell sequencing samples, and compared them according to the central and peripheral, human and mouse, and different T-cell categories (Fig. 1). Remarkably, a high proportion of single T cells expressing multiple TCR (>2) were identified. Furthermore, we performed traceability analysis on T cells with more than three TCR chains, which provides new insights for evaluating the proportion, molecular characteristics, mechanism of dual TCR T cells, and potential significance of allelic exclusion escape of V(D)J rearrangement.

## Results

### Expression of TCR chains in single T cells of human thymus

**Fig. 2:**
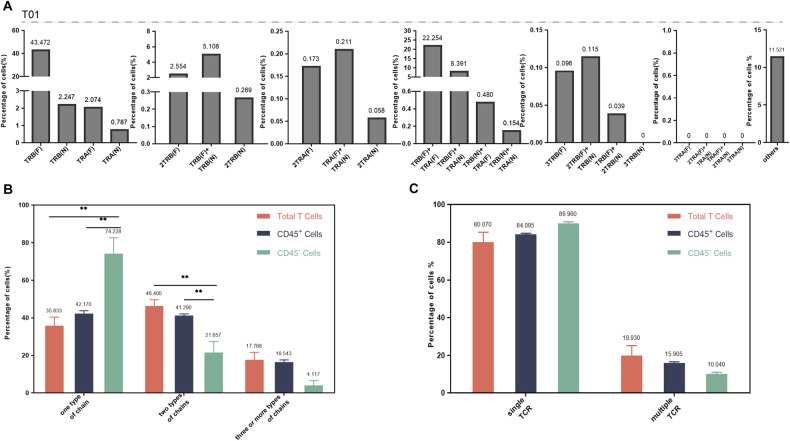
Single-cell immune profiling revealed multiple TCR chain patterns in human thymic single T cells. **A** The pattern and proportion of different TCR α and β chains detected in a single T-cell of thymus total T-cell (one sample, T01). **B** The difference of T cells with one, two, and three (or more) types of chains among total T cells, CD45^+^cells, and CD45^−^ cells in humans thymus. **C** The difference of T cells with single or multiple TCR among total T cells, CD45^+^cells, and CD45− cells in humans thymus. * is interpreted as *P* < 0.05. F functional, N non-functional.

The expression of TCR α and β chains of total T, CD45^+^, and CD45− cells category in the human thymus were analyzed. The number and proportion of different TCR α and β chains detected in a single T-cell were shown in Table S1, and we selected three representative samples from total T cells (Fig. 2A), CD45^+^ (Fig. S1A) and CD45− (Fig. S1B) cells category for visualization. Single cells of all samples mainly displayed 23 types of TCR α and (or) β chain, including functional (F) and non-functional (N) mRNA. TCR β (F) was the most common type of cell with only one chain, and TCR α (F)+ TCR β (F) was the main type of cells with two chains. The proportion of T cells with three different TCR chains was the lowest in all three groups (Fig. S1C). The proportion of cells with two chains detected in total T cells was the largest, accounting for 46.400%, significantly higher than CD45^+^ cells and CD45− cells. The proportion of CD45^+^ cells expressing one type of chain and two types of chains was similar (42.170 and 41.290%, respectively). CD45− cells expressing one type chain predominated, accounting for 74.228%, the proportion significantly higher than that of the other two groups (Fig. 2B).

The assembly of a TCR heterodimer consists of an α chain and a β chain. We assume that functional TCR contains at least one TCR α sequence (Functional) and one TCR β sequence (Functional). Although many TCR α and TCR β rearrangements were obtained from the human thymus, less than half of the cells that could assemble functional TCR (Table 1 and Fig. S1E). In total T cells, CD45^+^ and CD45^−^ cells samples, 49.407, 39.780, and 10.896% of the cells assembled to TCR, respectively. Notably, a certain proportion of cells with dual or more TCR were found in the three cell types. We screened functional T cells (with functional TCR) for further analysis, and the T cells with single TCR dominated in all three groups (Fig.S1D). There was no statistical difference in the proportion of cells with dual (or more) TCR in the total T cells, CD45^+^, and CD45^−^ cell subsets, which were 19.930, 15.905, and 10.040%, respectively (Fig. 2C).

**Table 1:** The number and proportion of cells resolved from single or multiple TCR.

Group	Sample	Login number or filename	Total number of T cells analyzed	The total number of unique sequences analyzed	Potential functional T cells (proportion%)	Single TCR/ Multiple TCR (%)
Human thymus	Total T cells (T01)	FCAImmP7851883	5208	8596	1668(32.028)	85.012/14.988
	Total T cells (T02)	T06_TH_TOT_VDJT_4	9864	22112	5971(60.533)	59.940/40.060
	Total T cells (T03)	T03_TH_TOT_VDJT_2	7079	12522	3007(42.478)	81.942/18.058
	Total T cells (T04)	FCAImmP7851887	3997	7181	1892(47.336)	83.510/16.490
	Total T cells (T05)	FCAImmP8105207	3107	5793	2009(64.660)	89.945/10.055
	Average				(49.407)	80.070/ 19.930
	CD45+ cells (P01)	FCAImmP7292943	5457	9362	2170(39.765)	82.811/17.189
	CD45+ cells (P02)	FCAImmP7292946	5102	9474	2125(41.650)	83.576/16.424
	CD45+ cells (P03)	FCAImmP7607591	2783	4785	1066(38.304)	83.865/16.135
	CD45+ cells (P04)	FCAImmP7607599	1736	3000	636(36.636)	86.792/13.208
	CD45+ cells (P05)	FCAImmP7607608	2468	4493	1050(42.545)	83.429/16.571
	Average				(39.780)	84.095/15.905
	CD45− cells (N01)	FCAImmP7607609	1877	2235	91(4.848)	89.011/10.989
	CD45− cells (N02)	FCAImmP7607600	844	1190	143(16.943)	90.909/9.091
	Average				(10.896)	89.960/10.040
Human peripheral blood	Frail individuals (R01)	GSM4750315	3457	7388	2167(62.684)	91.463/8.537
	Frail individuals (R02)	GSM4750312	993	2314	712(71.702)	93.961/6.039
	Frail individuals (R03)	GSM4750313	1102	2412	732(66.425)	89.481/10.519
	Frail individuals (R04)	GSM4750314	2041	4638	1421(69.623)	88.951/11.049
	Frail individuals (R05)	GSM4750316	980	2424	719(73.367)	92.629/7.371
	Average				(68.760)	91.297/8.703
	Young individuals (C01)	GSM4750317	3845	9310	2437(63.381)	90.562/9.438
	Young individuals (C02)	GSM4750318	4180	9563	2262(54.115)	89.920/10.080
	Young individuals (C03)	GSM4750319	4740	11575	2941(62.046)	89.629/10.371
	Average				(59.847)	90.037/9.963
	Old individuals (L01)	GSM5684311	6692	14197	4182(62.493)	86.227/13.773
	Old individuals (L02)	GSM4750323	3143	6721	2074(65.988)	88.042/11.958
	Old individuals (L03)	GSM4750325	5277	11824	3431(65.018)	87.205/12.795
	Old individuals (L04)	GSM5684309	5459	11556	3437(62.960)	85.569/14.431
	Old individuals (L05)	GSM5684310	6931	14769	4122(59.472)	84.619/15.381
	Average				(63.186)	86.332/13.668
Mouse peripheral lymphoid tissue	Lymph Nodes-Mouse1 (LN1)	GSM5172690	10141	22153	7555(74.500)	79.616/20.384
	Lymph Nodes-Mouse2 (LN2)	GSM5172691	10945	23463	7910(72.270)	79.897/20.103
	Lymph Nodes-Mouse3 (LN3)	GSM5172698	4270	8952	2654(62.155)	79.502/20.498
	Average				(69.642)	79.672/20.328
	Spleen-Mouse1 (S1)	GSM5172688	15024	30524	9337(62.147)	79.502/20.498
	Spleen-Mouse2 (S2)	GSM5172689	9362	20339	6973(74.482)	81.171/18.829
	Spleen-Mouse3 (S3)	GSM5172696	7362	15555	5383(73.119)	81.629/18.371
	Average				(69.916)	80.767/19.233
	Blood-Mouse1 (B1)	GSM5172686	7177	14709	5063(70.545)	82.895/17.105
	Blood-Mouse2 (B2)	GSM5172687	8276	17309	6058(73.200)	81.695/18.305
	Blood-Mouse3 (B3)	GSM5172694	3813	7703	2559(67.113)	81.595/18.405
	Average				(70.286)	82.062/17.938

### Expression of TCR chains in single T cells of human peripheral blood

**Fig. 3:**
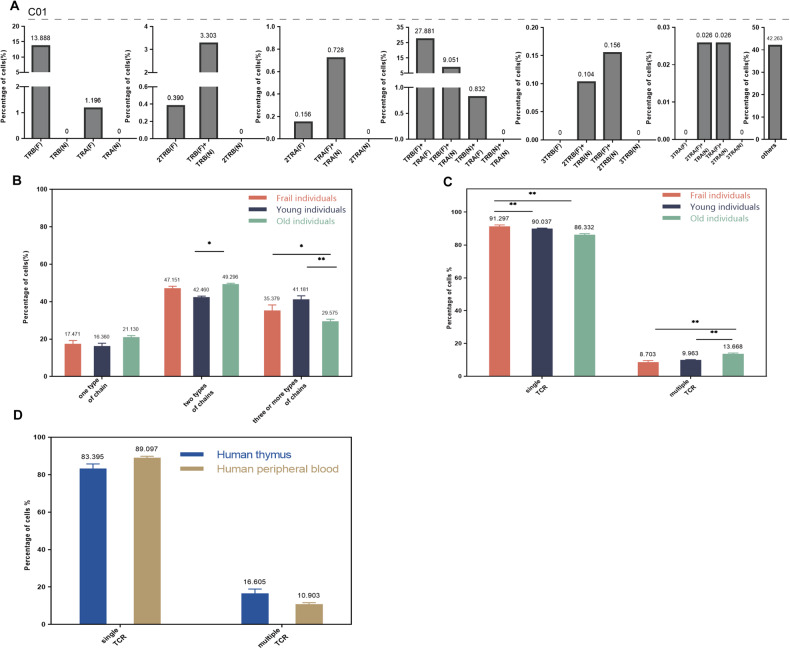
Single-cell immune profiling revealed multiple TCR chain patterns in human peripheral blood single T cells. **A** The pattern and proportion of different TCR α and β chains detected in a single T-cell of young peripheral blood (one sample, C01). **B** The difference of T cells with one, two, and three (or more) types of chains among young, frail, and old populations. **C** The difference of T cells with single or multiple TCR among young, frail, and old populations. **D** The difference in the proportion of single and multiple TCR T cells between human thymus and human peripheral blood. * is interpreted as *P* < 0.05. F functional, N non-functional.

In order to further understand the expression of TCR α and β chains in human peripheral T cells, we analyzed the peripheral blood single-cell sequencing data of frail, young, and old populations. Similarly, single or multiple TCR α and (or) β chains were detected in a single T-cell (Table S2 and Fig. 3A). TCR α (N) and TCR β (N) were not found in all samples, which was consistent with the characteristics of T cells after self-tolerance selection. T-cell expressing one type of chain was ~18% in three groups, and TCR β (F) was the domination. The number of T cells expressing two types of chains was most, and the combination of TCR α (F) and TCR β (F) had the highest frequency in all samples. The number of T cells expressing two types of TCR chains in all three populations was the highest (Fig. S2C) in all three populations. The number of T cells with three type chains was significantly different among frail (35.379%), young (41.181%), and old (29.575%) populations (Fig. 3B). Three representative samples from frail (Fig. S2A), young (Fig. 3A) and old (Fig. S2B) populations were selected for visualization.

The proportion of human peripheral blood T cells containing single TCR, multiple TCR, and abnormal TCR were also analyzed (Table S2). The proportion of cells with potential functional TCR detected in frail, adult young, and old individuals were 68.760, 59.847, and 63.186%, respectively (Table 1). The number of T cells with single TCR was significantly higher than that with abnormal TCR and multiple TCR in all samples (Fig. S2E). Among the three groups, the highest proportion of single TCR was found in frail individuals, the multiple TCR proportion was the highest in old individuals, and the proportion of abnormal TCR is relatively higher in young individuals. We screened functional T cells (excluding abnormal TCR T cells) for further analysis, and the T cells with single TCR dominated in all three groups (Fig. S2D). T cells with multiple TCR appeared more frequently (*P* < 0.05) in the old population compared to frail and young groups (Fig. 3C).

The comparative analysis was performed among the human thymus and human peripheral blood. In functional T cells, the proportion of single TCR T cells in peripheral blood was higher than that of thymus, while the proportion of dual TCR T cells was opposite (Fig. 3D). In all T cells, the proportion of abnormal TCR T cells in thymus was significantly higher than that in peripheral blood (Fig. S2F), and the proportion of single TCR T cells and dual TCR T cells were similar to that of functional T cells.

### Expression of TCR chain in single T-cell of mouse lymphoid tissue

**Fig. 4:**
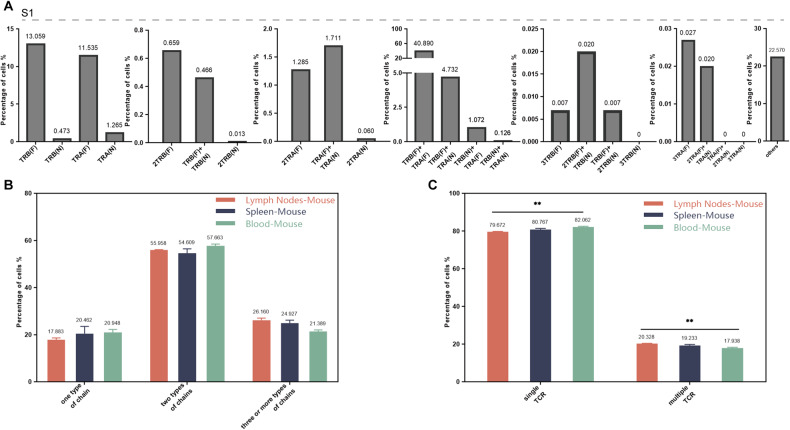
Single-cell immune profiling revealed multiple TCR chain patterns in mouse single T cells. **A** The pattern and proportion of different TCR α and β chains detected in a single T-cell of mouse spleen (one sample, S1). **B** The difference of T cells with one, two, and three (or more) types of chains among lymph nodes, spleen, and peripheral blood. **C** The difference of T cells with single or multiple TCR among lymph nodes, spleen, and peripheral blood.* is interpreted as *P* < 0.05. F functional, N non-functional.

As an important model animal, the single-cell sequencing data of lymph nodes, spleen, and peripheral blood of mice were also included in this study. Single T cells were found to express single or multiple TCR α and (or) β chains in all three mice tissues (Table S3). TCR β (F) was the domination mRNA in T-cells with one chain, the combination of TCR α (F) and TCR β (F) had the highest frequency in all samples, and T cells possess three TCR α(N) or three TCR β(N) were not found. Selected one representative sample from each group of lymph nodes (Fig. S3A), spleen (Fig. 4A), and peripheral blood (Fig. S3B) of mice for visualization.

T cells from lymph nodes, spleen, and peripheral blood mainly expressed two types of mRNA chains, and the proportion of one or three types of mRNA chains was both ~20% (Fig. S3C). T cells detected with one, two, or three or more chains was no significant difference among lymph nodes, spleen, and peripheral blood (Fig. 4B). The number of T cells with single TCR was significantly higher than that with abnormal TCR and multiple TCR in all three groups, and the proportion and trend of cells with single TCR, abnormal TCR and multiple TCR in the three groups were consistent (Fig. S3E). We screened functional T cells (excluding abnormal TCR T cells) for further analysis, and the T cells with single TCR dominated in all three groups (Fig. S3D). The proportion of dual (or more) TCR cells were 20.328, 19.233, and 17.938% in lymph nodes, spleen, and peripheral blood, respectively, and the proportion in lymph nodes was significantly higher than that in peripheral blood (Fig. 4C).

### Traceability of dual (or more) TCR

**Fig. 5:**
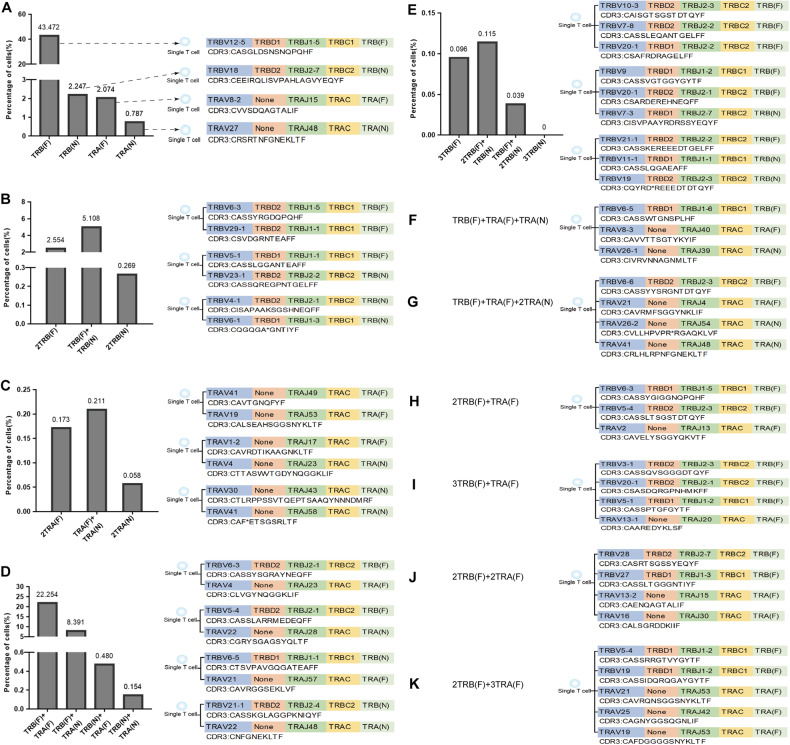
V(D)JC recombination patterns and CDR3 AA sequences in single T cells. **A** T-cell expressing single TCR α or TCR β; **B** T-cell expressing dual TCR β; **C** T-cell expressing dual TCR α; **D** T-cell expressing one TCR α and one TCR β; **E** T-cell expressing three TCR β; **F**, **G** T-cell expressing multiple chains and with one potential TCR; **H** T-cell with two potential TCR. **I** T-cell with three potential TCR. **J** T-cell with four potential TCR. **K** T-cell with five potential TCR. F functional, N non-functional.

Most TCR α and TCR β "V (D) J" mRNA sequences conform to the classical rules of 12RSS/23RSS rearrangement and allelic exclusion of TCR gene. However, single-cell expressed multiple TCR α and/or TCR β mRNA sequences were consistently observed in 34 single-cell sequencing samples, encompassing both central and peripheral immune organs, indicating that a single T-cell has three (or more) rearrangements and can undergo transcription and expression. Taking T01 sample of thymus as an example, we listed the V (D) JC family names and CDR3 AA sequences of eight representative TCR α and TCR β, including single TCR α or TCR β (Fig. 5A), dual TCR α or TCR β (Fig. 5 B, C), single TCR α and single TCR β (Fig. 5D), three TCR β (Fig. 5E), single TCR with multiple (≥3) TCR mRNA sequences (Fig. 5 F, G), dual TCR (Fig. 5H), three TCR (Fig. 5I), four TCR (Fig. 5J) and five TCR (Fig. 5K).

**Fig. 6:**
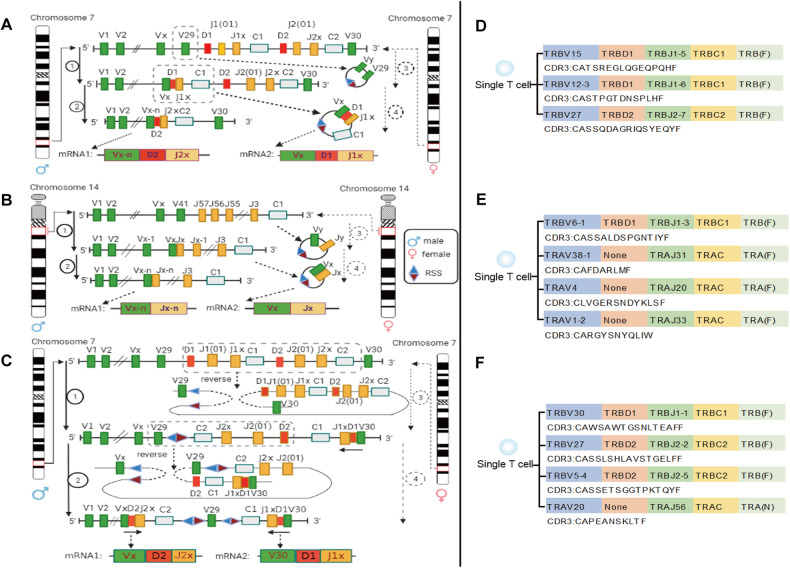
More than once TCR rearrangement on one chromosome. **A** The mechanism of two TCR β transcripts form one single chromosome by TREC circular DNA, and sequencing validation. **B** The mechanism of two TCR α transcripts form one single chromosome by TREC circular DNA. **C** The mechanism of two TCR β transcripts form one single chromosome by "reverse" recombinant. **D** The validation of mechanism A based on single T-cell sequencing data. **E** The validation of mechanism B based on single T-cell sequencing data. **F** The validation of mechanism C based on single T-cell sequencing data. F functional, N non-functional.

Based on the composition, location, RSS category, and direction of V (D) J gene at human and mouse TCR α and TCR β loci, we innovatively found that one of the three (or more) TCR β (or TCR α) in single T cells should be derived from the transcription of TREC circular DNA formed by secondary successful rearrangement. The first possible source of two TCR β transcripts on a single chromosome was shown in Fig. 6A, and the example of corresponding single T cells sequenced in the samples was shown in Fig. 6D. The possible source of two TCR α transcripts on a single chromosome was shown in Fig. 6B, and the example of corresponding single T cells sequenced was shown in Fig. 6E. Moreover, there is a reverse V gene (human V30 and mouse V31) on human and mouse TCR β loci. Therefore, the second possible source of two TCR β transcripts from a single chromosome was the "reverse" recombinant transcription (Fig. 6C) involving human V30 (or mouse V31), and the example of the corresponding single T-cell sequenced is shown in Fig. 6F.

### Dual TCR chains expression at protein level

To further understand the expression of multiple TCRα (or TCRβ) chains in single T cells, we conducted flow cytometry analyses on human peripheral blood and mouse lymphoid tissue. Among six volunteers, we detected the expression of dual TCRα chains in two individuals (TCR Vα7.2 and TCR Vα24), but no dual TCRβ chains were found (specifically, TCR Vβ8 and TCR Vβ13.1) (Table S4 and Fig. S4). In eleven mice from different age groups, we did not detect any T cells expressing dual TCRα chains, such as TCR Vα3.2, TCR Vα2, or TCR Vα11.1/11.2, or dual TCRβ chains, such as TCR Vβ5.1/5.2 or TCR Vβ13 (Table S4 and Fig. S5).

## Discussion

The classical theory that "one lymphocyte only expresses one specific antigen receptor" is supported by extensive experimental evidence, and its mechanism mainly depends on allelic exclusion [1]. However, the detailed mechanism of allelic exclusion has not been clarified [9, 10] and the lack of allelic exclusion has also been discovered by several teams, which a single T-cell expressed two TCR beta or alpha chains in humans and mice [22, 24, 26]. Single-cell sequencing can simultaneously sequence the mRNA of TCR α and TCR β chain from substantial single T cells. This innovative technology has provided new avenues for studying allelic exclusion escape and the proportion of dual TCR expression [29]. In this study, we leveraged single-cell sequencing data of human and mouse (central and peripheral) T cells shared by multiple laboratories to analyze the functional and non-functional TCR α and TCR β sequences of single T cells, including the proportion of one, two, and three or more types of chains and whether they can be paired as functional TCR. In addition, the proportion of single and multiple TCR in central and peripheral immune organs and their possible source mechanisms was also carried out [30, 31].

T cells with a"TCR α (F) + TCR β (F)" pattern are considered to have a functional TCR (Table 1). In all 34 samples, many T cells possess more than one "TCR α (F) + TCR β (F)" pattern, which could be assembled into dual (or more) TCR in single T cells (Fig. 5). In 12 thymus samples, the proportion of T cells with dual (or more) TCR accounts for more than 15% of the total number of T cells that can be paired to assemble TCR. The low proportion of T cells with dual (or more) TCR in CD45− and CD45^+^ subpopulations may be attributed to the early development of T cells, at which the TCR rearrangement of T cells has not been completed. The high proportion of dual (or more) TCR in total T cells (19.930%) indicates that TCR β (or TCR α) chains have experienced two or more functional rearrangements during the development of T cells, while more than two functional rearrangements of TCR α or TCR β chain in a single T-cell reveal that TCR rearrangements exist allelic exclusion escape.

Disease and aging will lead to a change in the normal functioning of the immune system [32]. It has been proved that aging-related thymic degeneration, repeated antigen contact, and inherent cell aging process will cause changes in T cells [33], and dual V beta-expressing cells expanded dramatically in the periphery with age, and such expanded cells had an activated phenotype [34]. In this study, the proportion of T cells with multiple TCR in an old group (13.668%) was significantly higher than that in young volunteers (9.963%) and frail individuals (8.703%). The increased T cells with dual (or more) TCR may be related to the disorder of rearrangement or abnormal tolerance selection after thymus recession.

The proportion of T cells with dual (or more) TCR in mouse lymph nodes (20.328%) was slightly higher than that in the spleen (19.233%) and peripheral blood (17.938%), which was consistent with Heath’s report that dual TCR was mainly found in mouse lymph nodes [35]. Moreover, the proportion of dual TCR cells found in this study is quite different from the existing reports (range from 1 to 30%) [34, 36, 37], indicating the complexity of dual TCR T cells and the necessity of further research. Interestingly, the proportion of T cells with multiple TCR in mouse peripheral lymphoid tissue is significantly higher than that in human peripheral blood, suggesting that the mechanism of allelic inclusion can be studied with the help of mice.

Theoretically, T cells express only one TCR after self-tolerance selection according to clonal selection theory. However, single-cell sequencing found a high proportion of multiple TCR α and TCR β chain types both in the central and peripheral immune tissues, and most of them could not assemble functional TCR. Therefore, we suggest that researchers should first classify and evaluate the cells with single TCR, multiple TCR, and abnormal TCR when performing the single T-cell sequencing data, and then unveil the relationship between their physiology and pathology to avoid deviation or even error in the results.

Single-cell sequencing showed a high proportion of TCR α or TCR β mRNA sequences which cannot be assembled into TCR. The possible reason is that single-cell sequencing technology failed to detect TCR α and TCR β mRNA in all single T cells at the same time, or failed to detect V (D) J sequences completely. Up to now, a similar phenomenon has been found in the research of single-cell sequencing applied to T or B-cell repertoire [16, 29–31]. Therefore, unpaired TCR chains should be separated in single-cell sequencing data and that should not be included in physiological or pathological correlation analysis, otherwise, TCR repertoire information will be expanded or distorted.

The characteristics of T-cell rearrangement in the thymus are as follows (TCR β example): (1) 1/3 of the functional TCR β that undergoing one rearrangement; (2) $\left( {1 - \frac{1}{3}} \right) \times \frac{1}{3}$ of the functional TCR β undergoing two rearrangements. The ratio of TCR β (F) and TCR β (N) of total T cells in the thymus in this study is basically consistent with the rearrangement rule. If both TCR β rearrangements fail, will "the third rearrangement" be started to save the apoptotic T cells? The research on this point is blank at present. There are TCR β (N) + TCR β (N) + TCR β (F) mRNA in a single cell, but the order of non-functional rearrangement and functional rearrangement cannot be determined.

T cells with three (or more) TCR β rearrangement events were identified in this study (Table S1-S3), which is inconsistent with the universally acknowledged TCR rearrangement rules: a single T-cell can only form two types of V (D) J combinations to participate in transcription. By analyzing RSS category, RSS direction, and the V (D) J gene position at TR locus of single T-cell which contains three (or more) TCR β or TCR α mRNA sequences, we creatively found that one of three TCR β (or TCR α) should be derived from the transcription of TREC circular DNA formed after the twice successful rearrangement of the same chromosome (Fig. 6). For TCR β chain, there is a reverse V gene (human V30 and mouse V31), so another possible mechanism is that human V30 (or mouse V31) participates in a "reverse" recombination and transcription. the forward and reverse V genes of TCR β locus participate in the rearrangement in single T-cells has been confirmed in V31 transgenic mice [38, 39]. This study is the first to propose a new mechanism of secondary (or more) TCR rearrangement on one chromosome, which provides a new idea for research on allelic exclusion escape. However, the underlying reasons for T cells undergoing three or more functional rearrangements of TCR α and β chain under physiological conditions remain unclear. Further research is needed to explore the potential significance of this phenomenon.

The proportion of mRNA level and protein level of dual TCR in a single T cell is reported differently in different laboratories [29, 40, 41]. Most of them are detected at mRNA level, but less at protein level. In this study, no "dual TCR β chain" T cells were detected at the protein level, but dual TCR α chain T cells could be detected in human peripheral blood samples. The high proportion of multiple TCR α or TCR β chain detected by single-cell sequencing requires more TCR alpha and beta chain antibodies for protein-level verification. Moreover, allelic exclusion may not only regulate on V(D)J gene rearrangement and transcription but also on the level of protein expression to ensure that most T cells ultimately express only one specific receptor.

In recent years, T cells with dual TCR have been found in autoimmune diseases [37, 42], aging [34], infection [43], malignant T-cell tumors [25], and GVHD [44]. With the help of single-cell sequencing technology, further analyze the mechanism and significance of allelic exclusion escape, which can provide new insights into the relationship between T cells with dual TCR and these diseases.

## Conclusion

This study found a high proportion of multiple TCR T cells by analyzing 34 single-cell sequencing data of humans and mice, including central and peripheral T cells, and proposed for the first time the new mechanism of secondary (or more) TCR rearrangement on one chromosome. Moreover, the single-cell sequencing data of T cells contained substantial sequences that could not be successfully assembled into functional TCR, suggesting that the data of single-cell sequencing should be classified according to single, multiple, and abnormal TCR when analyze the T-cell repertoire to avoid the deviation or even error of the repertoire on the physiological and pathological significance.

## Materials and methods

### Single-cell sequencing datasets acquisition

Three scRNA-seq datasets of T cells were downloaded from ArrayExpress and Gene Expression Omnibus (GEO) data repository, including three different T-cell categories (total, CD45^+^ and CD45− cells) of human thymus (EMTAB-8581), peripheral blood T cells of volunteers (GSE157007) under three different physiological conditions (frail, young and old) and T cells from three C57BL/6 mice lymph nodes, spleen and peripheral blood (GSE168944). The basic information of the above datasets was shown in Table 1. The total T cells (5 samples), CD45^+^ cells (5 samples), and CD45− cells (2 samples) of the human thymus are derived from thymic tissue during embryonic (9–39 weeks of pregnancy), neonatal (0–28 days), and adult (18–40 years of age) periods. The average age of the young (3 samples) and the olderly (5 samples) were 30.7 ± 10.0 years and 85.8 ± 11.1 years, respectively. According to the frailty index test, which includes chronic disease, disability, cognitive impairment, and stress index, elderly individuals aged 65 years or older with a score greater than 0.2 are defined as frailty (5 samples).

### Datasets preprocessing

The raw single-cell sequencing files of human thymus (T01-T05, P01-P05, N01-01), human peripheral blood (R01-R05, C01-C03, L01-L05) samples, and mouse peripheral tissue (LN1-LN3, S1-S3, B1-B2) samples were downloaded from Array Express database and NCBI database. Data quality control was performed in Microsoft Office Excel by screening and deleting sequences with the following four criteria: sequences in "is_cell" with "FALSE"; sequences in "high_confidence" with "FALSE"; sequences in "chain" that are not "TRA, TRB"; and sequences in "productive" with "None". Cells with high confidence, expressing TCR chains and defined as functional (F) or non-functional (N) can be used for subsequent analysis. Cells were classified according to the number of TCR chains expressed by a single cell. Then, combination analysis was performed according to the functionality of TCR chain include separate TCR α or TCR β chains and TCR α + TCR β. For example, cells with two TCR β chains were assembled into the following pattern: TCR β(F) + TCR β(F), TCR β(F) + TCR β(N), and TCR β(N) + TCR β(N), cells with one TCR α and one TCR β chain were assembled into the following pattern: TCR α(F) + TCR β(F), TCR α(F) + TCR β(N), TCR α(N) + TCR β(F) and TCR α(N) + TCR β(N). Finally, the cells were divided into 23 types according to the number of different TCR and the potential paired of TCR chain, which were (1) TCR β(F) (2) TCR β(N) (3) TCR α(F) (4) TCR α(N) (5) TCR β(F) + TCR β(F) (6) TCR β(F) + TCR β(N) (7) TCR β(N) + TCR β(N) (8) TCR α(F) + TCR α(F) (9) TCR α(F) + TCR α(N) (10) TCR α(N) + TCR α(N) (11) TCR β(F) + TCR α(F) (12) TCR β(F) + TCR α(N) (13) TCR β(N) + TCR α(F)(14) TCR β(N) + TCR α(N) (15) TCR β(F) + TCR β(F) + TCR β(F) (16) TCR β(F) + TCR β(F) + TCR β(N) (17) TCR β(F) + TCR β(N) + TCR β(N) (18) TCR β(N) + TCR β(N) + TCR β(N) (19) TCR α(F) + TCR α(F) + TCR α(F) (20) TCR α(F) + TCR α(F) + TCR α(N) (21) TCR α(F) + TCR α(N) + TCR α(N) (22) TCR α(N) + TCR α(N) + TCR α(N) and (23) others. Based on the number of different types of chains expressed in single T cells, they were divided into three groups: "one type of chain", "two types of chains", and "three or more types of chains". Based on the pairing of TRA and TRB chains, single T cells were statistically compared and analyzed in terms of "single TCR", "multiple TCR", and "abnormal TCR".

The functional V(D)J gene (no pseudogene family) recombined to form a fully transcribed mRNA sequence, which is called functional TCR α sequence or functional TCR β sequence. Functional T cells refer to a single T-cell that contains at least one TCR α (F) sequence and one TCR β (F) sequence. Non-functional T cells refer to (1) the absence of TCR α (F) and TCR β (F) sequences in a single T-cell; (2) Only TCR α (F) sequences in a single T-cell; (3) Only TCR β (F) sequences in a single T-cell.

“One type of chain" refers to the detection of only one TRA or TRB chain in a single T-cell, such as: TRA(F), TRA(N), and so on. That is, T cells that cannot be assembled into functional TCR at the transcriptome level. "Two types of chains" refer to the detection of two different TCR chains in a single T-cell, and the V(D)JC composition of these two sequences were different, such as: TRB(F) + TRA(F), TRB(F) + TRA(N), TRB(N) + TRA(F), TRB(N) + TRA(N) and so on. Among them, TRB(F) + TRA(F) is a cell type that can be assembled into a single functional TCR, and other combinations cannot be assembled into a functional TCR in a single cell. "Three or more types of chains" refers to the detection of three or more different chains in a single T-cell, and the V(D)JC composition of these three or more sequences were different, such as: TRB(F) + TRB(F) + TRB(F), TRB(F) + TRB(F) + TRB(N), TRB(F) + TRB(N) + TRB(N) and so on.

### Statistics of TCR expressed in single cells

Cells with a pair of TCR β (F) + TCR α (F) were defined as single TCR T cells, eg: TCR β (F) + TCR α (F)+nTCR α (N)+nTCR β (N). Three or more functional TCR chains expressed by a single cell can be combined into different patterns of TCR β (F) + TCR α (F), and these cells were defined as multiple TCR cells, eg: TCR β(F) + TCR α(F)+nTCR β(F/N)+nTCR α(F/N). The T cells without any combination of TCR β (F) + TCR α (F) were defined as abnormal TCR cells. Then, the analysis of the proportion and difference of cells with single TCR, multiple TCR, and abnormal TCR was performed among all samples.

### Traceability of multiple TCR

T cells expressing three or more TCR chains were identified in all samples, which is inconsistent with the generally recognized principle of allosteric exclusion. Therefore, we screened the cells expressing three TCR chains and their sequences for traceability analysis according to TCR rearrangement rule, including RSS category, RSS direction, and the V(D)J gene position at TR locus.

### Flow cytometry

Peripheral blood samples were collected from six healthy volunteers. Additionally, spleen, lymph nodes, and thymus samples were collected from eleven BALB/c mice (Table S5). This experiment was approved by the Ethics Committee of Zunyi Medical University and with the informed consent of healthy volunteers (ZMU [2021]1-022). Single-cell suspensions of lymphocytes from human peripheral blood were incubated with red blood cell lysis buffer (0.7 M NaCl and 17 mM Tris HCl). Cells were washed with FACS staining buffer (PBS containing 0.5% BSA) and stained with the following Biolgend antibodies: APC-TCR Vα7.2 (351707), PC5.5-TCR Vα24 (360003), FITC-CD3 (317305), PE-TCR Vβ13.1 (362409), APC-TCR Vβ8 (348105), APC-TCRβ5.1/5.2 (139505), PE-TCR Vβ13 (140703), APC-TCR Vα2 (127809), FITC-TCR Vα3.2 (135403), PE-TCR Vα11.1/11.2 (139903) and PC5.5-CD3(100217). Cells were stained in FACS staining buffer. All flow cytometry analyses were performed on a Partec PAS. Data were analyzed with the help of FlowJo software.

### Statistical analysis

Statistical analysis was performed with IBM SPSS Statistics 26 software. Two-tailed *t* tests were suitable for comparison analysis between two groups of data that conform to the normal distribution, one-way ANOVA was applied for three or more independent groups with normally distributed and homogenous variance data. While data that were not normally distributed were analyzed with the non-parametric Mann–Whitney test and Kruskal–Wallis test. Data were represented as means ± SEM. Significance levels were labeled as *P < 0.05, **P < 0.01. GraphPad Prism software and Adobe Illustrator software were used for data visualization.

## Supplementary information

Supplemental file

A reproducibility checklist

## Data Availability

All data are available in the main text or the supplementary materials.

## References

[1] Burnet FM (1959). The clonal selection theory of acquired immunity[M].

[2] Schlissel M (2002). Allelic exclusion of immunoglobulin gene rearrangement and expression: why and how?. Semin Immunol.

[3] Aifantis I, Buer J, von Boehmer H, Azogui O (1997). Essential role of the pre-T cell receptor in allelic exclusion of the T cell receptor beta locus. Immunity.

[4] Michie AM, Soh JW, Hawley RG, Weinstein IB, Zuniga-Pflucker JC (2001). Allelic exclusion and differentiation by protein kinase C-mediated signals in immature thymocytes. Proc Natl Acad Sci USA.

[5] Khor B, Sleckman BP (2002). Allelic exclusion at the TCRbeta locus. Curr Opin Immunol.

[6] Eyquem S, Chemin K, Fasseu M, Bories JC (2004). The Ets-1 transcription factor is required for complete pre-T cell receptor function and allelic exclusion at the T cell receptor beta locus. Proc Natl Acad Sci USA.

[7] Wu GS, Bassing CH (2020). Inefficient V(D)J recombination underlies monogenic T cell receptor beta expression. Proc Natl Acad Sci USA.

[8] Wu GS, Yang-Iott KS, Klink MA, Hayer KE, Lee KD, Bassing CH (2020). Poor quality Vβ recombination signal sequences stochastically enforce TCRβ allelic exclusion. J Exp Med.

[9] Vettermann C, Schlissel MS (2010). Allelic exclusion of immunoglobulin genes: models and mechanisms. Immunol Rev.

[10] Outters P, Jaeger S, Zaarour N, Ferrier P (2015). Long-range control of V(D)J recombination & allelic exclusion: modeling views. Adv Immunol.

[11] Makela O, Nossal GJ (1961). Study of antibody-producing capacity of single cells by bacterial adherence and immobilization. J Immunol.

[12] ten Boekel E, Melchers F, Rolink AG (1998). Precursor B cells showing H chain allelic inclusion display allelic exclusion at the level of pre-B cell receptor surface expression. Immunity.

[13] Casellas R, Zhang Q, Zheng NY, Mathias MD, Smith K, Wilson PC (2007). Igkappa allelic inclusion is a consequence of receptor editing. J Exp Med.

[14] Velez MG, Kane M, Liu S, Gauld SB, Cambier JC, Torres RM (2007). Ig allotypic inclusion does not prevent B cell development or response. J Immunol.

[15] Fournier EM, Velez MG, Leahy K, Swanson CL, Rubtsov AV, Torres RM (2012). Dual-reactive B cells are autoreactive and highly enriched in the plasmablast and memory B cell subsets of autoimmune mice. J Exp Med.

[16] Shi Z, Zhang Q, Yan H, Yang Y, Wang P, Zhang Y (2019). More than one antibody of individual B cells revealed by single-cell immune profiling. Cell Discov.

[17] Jiwani S, Bornhost J, Alapat D (2015). Biphenotypic plasma cell myeloma: two cases of plasma cell neoplasm with a coexpression of kappa and lambda light chains. Int J Clin Exp Pathol.

[18] Jiang AS, Wu Z, Wei EX, Ni H, You B, Yang T (2018). Plasma cell myeloma with dual expression of kappa and lambda light chains. Int J Clin Exp Pathol.

[19] Matsuoka R, Sakamoto N, Kato T, Chiba S, Noguchi M (2021). A case of solitary plasmacytoma of bone showing co-expression of both immunoglobulin light chains. Eur J Med Res.

[20] Peterson JN, Boackle SA, Taitano SH, Sang A, Lang J, Kelly M (2022). Elevated detection of dual antibody B cells identifies lupus patients with B cell-reactive VH4-34 autoantibodies. Front Immunol.

[21] Ahmed R, Omidian Z, Giwa A, Cornwell B, Majety N, Bell DR (2019). A public BCR present in a unique dual-receptor-expressing lymphocyte from type 1 diabetes patients encodes a potent T cell autoantigen. Cell.

[22] Malissen M, Trucy J, Letourneur F, Rebaï N, Dunn DE, Fitch FW (1988). A T cell clone expresses two T cell receptor alpha genes but uses one alpha beta heterodimer for allorecognition and self MHC-restricted antigen recognition. Cell.

[23] Matsuoka R, Sakamoto N, Kato T, Chiba S, Noguchi M (1988). Evidence for expression of two distinct T cell receptor beta-chain transcripts in a human diphtheria toxoid-specific T cell clone. J Immunol.

[24] Padovan E, Casorati G, Dellabona P, Meyer S, Brockhaus M, Lanzavecchia A (1993). Expression of two T cell receptor alpha chains: dual receptor T cells. Science.

[25] Hinz T, Weidmann E, Kabelitz D (2001). Dual TCR-expressing T lymphocytes in health and disease. Int Arch Allergy Immunol.

[26] Gavin MA, Rudensky AY (2002). Dual TCR T cells: gaining entry into the periphery. Nat Immunol.

[27] Brady BL, Steinel NC, Bassing CH (2010). Antigen receptor allelic exclusion: an update and reappraisal. J Immunol.

[28] Balakrishnan A, Morris GP (2016). The highly alloreactive nature of dual TCR T cells. Curr Opin Organ Transpl.

[29] Stubbington MJT, Lönnberg T, Proserpio V, Clare S, Speak AO, Dougan G (2016). T cell fate and clonality inference from single-cell transcriptomes. Nat Methods.

[30] Park JE, Botting RA, Domínguez Conde C, Popescu DM, Lavaert M, Kunz DJ (2020). A cell atlas of human thymic development defines T cell repertoire formation. Science.

[31] Luo OJ, Lei W, Zhu G, Ren Z, Xu Y, Xiao C (2022). Multidimensional single-cell analysis of human peripheral blood reveals characteristic features of the immune system landscape in aging and frailty. Nat Aging.

[32] Franceschi C, Garagnani P, Parini P, Giuliani C, Santoro A (2018). Inflammaging: a new immune-metabolic viewpoint for age-related diseases. Nat Rev Endocrinol.

[33] Elyahu Y, Hekselman I, Eizenberg-Magar I, Berner O, Strominger I, Schiller M (2019). Aging promotes reorganization of the CD4 T cell landscape toward extreme regulatory and effector phenotypes. Sci Adv.

[34] Balomenos D, Balderas RS, Mulvany KP, Kaye J, Kono DH, Theofilopoulos AN (1995). Incomplete T cell receptor V beta allelic exclusion and dual V beta-expressing cells. J Immunol.

[35] Heath WR, Carbone FR, Bertolino P, Kelly J, Cose S, Miller JF (1995). Expression of two T cell receptor alpha chains on the surface of normal murine T cells. Eur J Immunol.

[36] Munthe LA, Blichfeldt E, Sollien A, Dembic Z, Bogen B (1996). T cells with two Tcrbeta chains and reactivity to both MHC/idiotypic peptide and superantigen. Cell Immunol.

[37] Corthay A, Nandakumar KS, Holmdahl R (2001). Evaluation of the percentage of peripheral T cells with two different T cell receptor alpha-chains and of their potential role in autoimmunity. J Autoimmun.

[38] Khor B, Sleckman BP (2005). Intra- and inter-allelic ordering of T cell receptor beta chain gene assembly. Eur J Immunol.

[39] Lee KD, Bassing CH (2020). Two successive inversional Vβ rearrangements on a single Tcrb allele can contribute to the TCRβ repertoire. J Immunol.

[40] Dash P, McClaren JL, Oguin TH, Rothwell W, Todd B, Morris MY (2011). Paired analysis of TCRα and TCRβ chains at the single-cell level in mice. J Clin Invest.

[41] Steinel NC, Brady BL, Carpenter AC, Yang-Iott KS, Bassing CH (2010). Posttranscriptional silencing of VbetaDJbetaCbeta genes contributes to TCRbeta allelic exclusion in mammalian lymphocytes. J Immunol.

[42] Sarukhan A, Garcia C, Lanoue A, von Boehmer H (1998). Allelic inclusion of T cell receptor alpha genes poses an autoimmune hazard due to low-level expression of autospecific receptors. Immunity.

[43] Ji Q, Perchellet A, Goverman JM (2010). Viral infection triggers central nervous system autoimmunity via activation of CD8+ T cells expressing dual TCRs. Nat Immunol.

[44] Vincent BG, Serody JS (2013). One is better than two: TCR pairing and GVHD. Sci Transl Med.

