# Supplementary material for: scRNA-seq revealed the special TCR β & α V(D)J allelic inclusion rearrangement and the high proportion dual (or more) TCR-expressing cells
Source: Cell Death Dis. 2023 Jul 31;14(7):487. doi: 10.1038/s41419-023-06004-7 (PMC10390570; doi:10.1038/s41419-023-06004-7)
Supplement: Supplementary file 1 — Supplemental file [file 41419_2023_6004_MOESM1_ESM.docx]

Table S1 Categories and proportions of TRA and TRB from human thymus samples

| Samples | Total T Cells (%) | | | | | | CD45^+^ Cells (%) | | | | | | CD45^-^ Cells (%) | | |
| --- | --- | --- | --- | --- | --- | --- | --- | --- | --- | --- | --- | --- | --- | --- | --- |
| Category | T01 | T02 | T03 | T04 | T05 | Average | P01 | P02 | P03 | P04 | P05 | Average | N01 | N02 | Average |
| TRB(F) | 43.472 | 22.638 | 38.508 | 36.978 | 21.79 | 32.677 | 40.535 | 35.908 | 43.478 | 40.38 | 37.561 | 39.572 | 53.65 | 46.564 | 50.107 |
| TRB(N) | 2.247 | 0.041 | 0.537 | 0.951 | 0.805 | 0.916 | 1.338 | 0.941 | 1.581 | 1.152 | 0.689 | 1.14 | 28.556 | 18.601 | 23.579 |
| TRA(F) | 2.074 | 0.183 | 1.116 | 1.051 | 4.828 | 1.85 | 2.529 | 0.608 | 0.827 | 1.152 | 0.648 | 1.153 | 0.213 | 0.474 | 0.344 |
| TRA(N) | 0.787 | 0.02 | 0.353 | 0.175 | 0.612 | 0.389 | 0.587 | 0.196 | 0.108 | 0.346 | 0.284 | 0.304 | 0.16 | 0.237 | 0.199 |
| 2TRB(F) | 2.554 | 5.708 | 3.899 | 1.701 | 0.515 | 2.875 | 1.649 | 3.077 | 2.012 | 2.934 | 1.378 | 2.21 | 1.332 | 1.659 | 1.496 |
| TRB(F)+TRB(N) | 5.108 | 1.592 | 3.348 | 4.003 | 0.708 | 2.952 | 4.398 | 6.547 | 4.851 | 5.876 | 4.336 | 5.202 | 5.97 | 6.754 | 6.362 |
| 2TRB(N) | 0.269 | 0 | 0 | 0.125 | 0.032 | 0.085 | 0.037 | 0.079 | 0.431 | 0.346 | 0.041 | 0.187 | 2.77 | 1.777 | 2.274 |
| 2TRA(F) | 0.173 | 0.031 | 0.113 | 0.1 | 0.354 | 0.154 | 0.238 | 0.079 | 0.144 | 0.289 | 0.162 | 0.182 | 0 | 0 | 0 |
| TRA(F)+TRA(N) | 0.211 | 0.02 | 0.113 | 0.175 | 0.483 | 0.2 | 0.33 | 0.039 | 0.143 | 0.058 | 0.203 | 0.155 | 0.053 | 0.119 | 0.086 |
| 2TRA(N) | 0.058 | 0 | 0.014 | 0 | 0 | 0.014 | 0.055 | 0 | 0 | 0 | 0.041 | 0.019 | 0 | 0 | 0 |
| TRB(F)+TRA(F) | 22.254 | 31.488 | 30.075 | 32.349 | 52.012 | 33.636 | 27.085 | 25.01 | 24.398 | 24.828 | 26.661 | 25.596 | 3.676 | 11.848 | 7.762 |
| TRB(F)+TRA(N) | 8.391 | 5.099 | 6.95 | 5.704 | 4.249 | 6.079 | 6.194 | 7.546 | 5.498 | 7.892 | 8.874 | 7.201 | 1.971 | 4.621 | 3.296 |
| TRB(N)+TRA(F) | 0.48 | 0.041 | 0.283 | 0.3 | 0.483 | 0.317 | 0.495 | 0.255 | 0.647 | 0.461 | 0.284 | 0.428 | 0.053 | 0.355 | 0.204 |
| TRB(N)+TRB(N) | 0.154 | 0.041 | 0.042 | 0.1 | 0.097 | 0.087 | 0.183 | 0.157 | 0.036 | 0.173 | 0 | 0.11 | 0 | 0.355 | 0.178 |
| 3TRB(F) | 0.096 | 0.771 | 0.155 | 0.025 | 0 | 0.209 | 0 | 0.079 | 0 | 0.115 | 0 | 0.039 | 0.053 | 0 | 0.027 |
| 2TRB(F)+TRB(N) | 0.115 | 0.446 | 0.24 | 0.125 | 0 | 0.185 | 0.128 | 0.098 | 0.18 | 0.115 | 0 | 0.104 | 0 | 0 | 0 |
| TRB(F)+2TRB(N) | 0.039 | 0 | 0.028 | 0.025 | 0 | 0.018 | 0.073 | 0.02 | 0.036 | 0.173 | 0.041 | 0.069 | 0 | 0.119 | 0.06 |
| 3TRB(N) | 0 | 0 | 0 | 0 | 0 | 0 | 0 | 0 | 0 | 0 | 0 | 0 | 0.107 | 0 | 0.054 |
| 3TRA(F) | 0 | 0 | 0 | 0 | 0 | 0 | 0 | 0 | 0 | 0 | 0 | 0 | 0 | 0 | 0 |
| 2TRA(F)+TRA(N) | 0 | 0 | 0 | 0 | 0.032 | 0.006 | 0 | 0 | 0 | 0 | 0 | 0 | 0 | 0 | 0 |
| TRA(F)+2TRA(N) | 0 | 0 | 0 | 0 | 0 | 0 | 0 | 0 | 0 | 0 | 0 | 0 | 0 | 0 | 0 |
| 3TRA(N) | 0 | 0 | 0 | 0 | 0 | 0 | 0 | 0 | 0 | 0 | 0 | 0 | 0 | 0 | 0 |
| Others | 11.521 | 31.883 | 14.226 | 16.112 | 13.003 | 17.349 | 14.148 | 19.365 | 15.631 | 13.712 | 18.801 | 16.331 | 1.438 | 6.516 | 3.977 |
| Total | 100 | 100 | 100 | 100 | 100 | 100 | 100 | 100 | 100 | 100 | 100 | 100 | 100 | 100 | 100 |
| Single TCR | 27.228 | 36.283 | 34.807 | 39.529 | 58.159 | 39.201 | 32.931 | 34.810 | 32.124 | 31.798 | 35.494 | 33.431 | 4.315 | 15.403 | 9.859 |
| Multiple TCR | 4.800 | 24.250 | 7.670 | 7.805 | 6.501 | 10.100 | 6.835 | 6.841 | 6.181 | 4.839 | 7.051 | 6.349 | 0.532 | 1.540 | 1.036 |
| Abnormal TCR | 67.975 | 39.469 | 57.523 | 52.665 | 35.342 | 50.700 | 60.236 | 58.352 | 61.696 | 63.363 | 57.458 | 60.221 | 95.155 | 83.057 | 89.106 |
| Total | 100.00 | 100.00 | 100.00 | 100.00 | 100.00 | 100.00 | 100.00 | 100.00 | 100.00 | 100.00 | 100.00 | 100.00 | 100.00 | 100.00 | 100.00 |
|  |  |  |  |  |  |  |  |  |  |  |  |  |  |  |  |
| One type of chain | 48.580 | 22.882 | 40.514 | 39.155 | 28.035 | 35.833 | 44.989 | 37.653 | 45.994 | 43.030 | 39.182 | 42.170 | 82.579 | 65.876 | 74.228 |
| Two types of chains | 39.652 | 44.020 | 44.837 | 44.557 | 58.933 | 46.400 | 40.664 | 42.789 | 38.160 | 42.857 | 41.980 | 41.290 | 15.825 | 27.488 | 21.657 |
| Three or more types of chains | 11.771 | 33.100 | 14.649 | 16.287 | 13.035 | 17.768 | 14.349 | 19.562 | 15.847 | 14.115 | 18.842 | 16.543 | 1.598 | 6.635 | 4.117 |
| Total | 100.00 | 100.00 | 100.00 | 100.00 | 100.00 | 100.00 | 100.00 | 100.00 | 100.00 | 100.00 | 100.00 | 100.00 | 100.00 | 100.00 | 100.00 |

Single TCR includes the following cases: TRB(F)+TRA(F)+xTRB(N)+yTRA(N); Multiple TCR includes the following cases TRB(F)+TRA(F)+TRB/TRA(F)

+x1TRB(F)+x2TRB(N)+y1TRA(F)+y2TRA(N);Abnormal TCR includes the following cases xTRB(F)、xTRB(N)、yTRA(F)、yTRA(N)、xTRB(N)+yTRA(N)、

xTRB(F)+yTRA(N)、xTRB(N)+yTRA(F)(x,x1,x2,y,y1,y2 as an integer). F: functional; N: non-functional.

Table S2 Categories and proportions of TRA and TRB from human peripheral blood samples

| Sample | Frail individuals (%) | | | | | | Young individuals (%) | | | | Old individuals (%) | | | | | |
| --- | --- | --- | --- | --- | --- | --- | --- | --- | --- | --- | --- | --- | --- | --- | --- | --- |
| Category | R01 | R02 | R03 | R04 | R05 | Average | C01 | C02 | C03 | Average | L01 | L02 | L03 | L04 | L05 | Average |
| TRB(F) | 21.261 | 14.804 | 19.782 | 17.296 | 11.735 | 16.976 | 13.888 | 18.278 | 13.987 | 15.384 | 19.022 | 20.331 | 18.003 | 19.784 | 20.762 | 19.58 |
| TRB(N) | 0 | 0 | 0 | 0 | 0 | 0 | 0 | 0 | 0 | 0 | 0 | 0 | 0 | 0 | 0 | 0 |
| TRA(F) | 0.694 | 0.403 | 0.635 | 0.441 | 0.306 | 0.496 | 1.196 | 1.077 | 0.654 | 0.976 | 2.242 | 0.573 | 0.398 | 3.078 | 1.457 | 1.55 |
| TRA(N) | 0 | 0 | 0 | 0 | 0 | 0 | 0 | 0 | 0 | 0 | 0 | 0 | 0 | 0 | 0 | 0 |
| 2TRB(F) | 0.289 | 0 | 0.817 | 0.245 | 0.306 | 0.331 | 0.39 | 3.852 | 0.38 | 1.541 | 0.433 | 0.446 | 0.701 | 0.55 | 2.323 | 0.891 |
| TRB(F)+TRB(N) | 2.864 | 3.323 | 2.813 | 4.214 | 3.98 | 3.439 | 3.303 | 4.019 | 4.051 | 3.791 | 4.02 | 3.341 | 4.7 | 3.902 | 3.953 | 3.983 |
| 2TRB(N) | 0 | 0 | 0 | 0 | 0 | 0 | 0 | 0 | 0 | 0 | 0 | 0 | 0 | 0 | 0 | 0 |
| 2TRA(F) | 0.058 | 0 | 0 | 0 | 0.102 | 0.032 | 0.156 | 0.191 | 0.021 | 0.123 | 0.687 | 0.032 | 0.057 | 0.513 | 0.058 | 0.269 |
| TRA(F)+TRA(N) | 0.058 | 0.101 | 0.181 | 0.049 | 0 | 0.078 | 0.728 | 0.431 | 0.317 | 0.492 | 0.359 | 0.096 | 0.152 | 0.403 | 0.419 | 0.286 |
| 2TRA(N) | 0 | 0 | 0 | 0 | 0 | 0 | 0 | 0 | 0 | 0 | 0 | 0 | 0 | 0 | 0 | 0 |
| TRB(F)+TRA(F) | 37.374 | 37.664 | 38.656 | 37.335 | 32.449 | 36.696 | 27.881 | 25.12 | 25.612 | 26.204 | 38.105 | 39.58 | 35.247 | 37.498 | 35.175 | 37.121 |
| TRB(F)+TRA(N) | 8.042 | 6.042 | 5.445 | 5.096 | 5.816 | 6.088 | 9.051 | 9.091 | 10.443 | 9.528 | 5.992 | 6.077 | 6.86 | 3.975 | 6.68 | 5.917 |
| TRB(N)+TRA(F) | 0.665 | 0.302 | 0.817 | 0.245 | 0.408 | 0.487 | 0.832 | 0.646 | 0.865 | 0.781 | 1.285 | 0.318 | 0.379 | 1.429 | 0.736 | 0.829 |
| TRB(N)+TRB(N) | 0 | 0 | 0 | 0 | 0 | 0 | 0 | 0 | 0 | 0 | 0 | 0 | 0 | 0 | 0 | 0 |
| 3TRB(F) | 0 | 0 | 0 | 0 | 0 | 0 | 0 | 0 | 0 | 0 | 0 | 0 | 0 | 0 | 0 | 0 |
| 2TRB(F)+TRB(N) | 0.058 | 0 | 0.272 | 0 | 0 | 0.066 | 0.104 | 1.1 | 0.021 | 0.408 | 0.03 | 0.095 | 0.076 | 0.128 | 0.274 | 0.121 |
| TRB(F)+2TRB(N) | 0.232 | 0.101 | 0.635 | 0.098 | 0.306 | 0.274 | 0.156 | 0.359 | 0.316 | 0.277 | 0.299 | 0.064 | 0.284 | 0.183 | 0.216 | 0.209 |
| 3TRB(N) | 0 | 0 | 0 | 0 | 0 | 0 | 0 | 0 | 0 | 0 | 0 | 0 | 0 | 0 | 0 | 0 |
| 3TRA(F) | 0 | 0 | 0 | 0 | 0 | 0 | 0 | 0 | 0 | 0 | 0 | 0 | 0 | 0 | 0 | 0 |
| 2TRA(F)+TRA(N) | 0 | 0 | 0 | 0 | 0 | 0 | 0.026 | 0.048 | 0 | 0.025 | 0.015 | 0 | 0 | 0 | 0 | 0.003 |
| TRA(F)+2TRA(N) | 0 | 0 | 0 | 0 | 0 | 0 | 0.026 | 0 | 0.021 | 0.016 | 0.015 | 0 | 0 | 0.037 | 0 | 0.01 |
| 3TRA(N) | 0 | 0 | 0 | 0 | 0 | 0 | 0 | 0 | 0 | 0 | 0 | 0 | 0 | 0 | 0 | 0 |
| Others | 28.407 | 37.262 | 29.947 | 34.983 | 44.593 | 35.038 | 42.263 | 35.79 | 43.313 | 40.455 | 27.496 | 29.05 | 33.144 | 28.523 | 27.948 | 29.232 |
| Total | 100 | 100 | 100 | 100 | 100 | 100 | 100 | 100 | 100 | 100 | 100 | 100 | 100 | 100 | 100 | 100 |
| Single TCR | 57.334 | 67.373 | 59.436 | 61.931 | 67.960 | 62.807 | 57.400 | 48.658 | 55.612 | 53.890 | 53.885 | 58.097 | 56.699 | 53.875 | 50.325 | 54.580 |
| Multiple TCR | 5.351 | 4.331 | 6.987 | 7.693 | 5.408 | 5.954 | 5.982 | 5.455 | 6.436 | 5.958 | 8.607 | 7.890 | 8.319 | 9.086 | 9.148 | 8.610 |
| Abnormal TCR | 37.316 | 28.297 | 33.575 | 30.379 | 26.632 | 31.240 | 36.618 | 45.887 | 37.954 | 40.153 | 37.507 | 34.014 | 34.983 | 37.042 | 40.528 | 36.815 |
| Total | 100.00 | 100.00 | 100.00 | 100.00 | 100.00 | 100.00 | 100.00 | 100.00 | 100.00 | 100.00 | 100.00 | 100.00 | 100.00 | 100.00 | 100.00 | 100.00 |
|  |  |  |  |  |  |  |  |  |  |  |  |  |  |  |  |  |
| One type of chain | 21.955 | 15.207 | 20.417 | 17.737 | 12.041 | 17.471 | 15.084 | 19.355 | 14.641 | 16.360 | 21.264 | 20.904 | 18.401 | 22.862 | 22.219 | 21.130 |
| Two types of chains | 49.350 | 47.432 | 48.729 | 47.184 | 43.061 | 47.151 | 42.341 | 43.350 | 41.689 | 42.460 | 50.881 | 49.890 | 48.096 | 48.270 | 49.344 | 49.296 |
| Three or more types of chains | 28.697 | 37.363 | 30.854 | 35.081 | 44.899 | 35.379 | 42.575 | 37.297 | 43.671 | 41.181 | 27.855 | 29.209 | 33.504 | 28.871 | 28.438 | 29.575 |
| Total | 100.00 | 100.00 | 100.00 | 100.00 | 100.00 | 100.00 | 100.00 | 100.00 | 100.00 | 100.00 | 100.00 | 100.00 | 100.00 | 100.00 | 100.00 | 100.00 |

Single TCR includes the following cases: TRB(F)+TRA(F)+xTRB(N)+yTRA(N); Multiple TCR includes the following cases TRB(F)+TRA(F)+TRB/TRA(F)

+x1TRB(F)+x2TRB(N)+y1TRA(F)+y2TRA(N);Abnormal TCR includes the following cases xTRB(F)、xTRB(N)、yTRA(F)、yTRA(N)、xTRB(N)+yTRA(N)、

xTRB(F)+yTRA(N)、xTRB(N)+yTRA(F)(x,x1,x2,y,y1,y2 as an integer). F: functional; N: non-functional.

Table S3 Categories and proportions of TRA and TRB in peripheral blood T cells from mouse lymph nodes and spleens

| Sample | Lymph Nodes-Mouse (%) | | | | Spleen-Mouse (%) | | | | Blood-Mouse (%) | | | |
| --- | --- | --- | --- | --- | --- | --- | --- | --- | --- | --- | --- | --- |
| category | LN1 | LN2 | LN3 | Average | S1 | S2 | S3 | Average | B1 | B2 | B3 | Average |
| TRB(F) | 8.441 | 9.868 | 12.81 | 10.373 | 13.059 | 8.246 | 12.021 | 11.109 | 12.707 | 11.672 | 15.526 | 13.302 |
| TRB(N) | 0.276 | 0.292 | 0.656 | 0.408 | 0.473 | 0.171 | 0.272 | 0.305 | 0.209 | 0.35 | 0.367 | 0.309 |
| TRA(F) | 7.07 | 7.41 | 5.222 | 6.567 | 11.535 | 7.135 | 6.208 | 8.293 | 6.939 | 6.319 | 7.081 | 6.78 |
| TRA(N) | 0.552 | 0.676 | 0.375 | 0.534 | 1.265 | 0.566 | 0.435 | 0.755 | 0.585 | 0.616 | 0.472 | 0.558 |
| 2TRB(F) | 0.276 | 0.292 | 0.609 | 0.392 | 0.659 | 0.256 | 0.598 | 0.504 | 0.502 | 0.35 | 1.049 | 0.634 |
| TRB(F)+TRB(N) | 0.404 | 0.402 | 0.609 | 0.472 | 0.466 | 0.342 | 0.435 | 0.414 | 0.307 | 0.483 | 0.577 | 0.456 |
| 2TRB(N) | 0 | 0.018 | 0 | 0.006 | 0.013 | 0.021 | 0 | 0.011 | 0 | 0 | 0 | 0 |
| 2TRA(F) | 1.006 | 1.261 | 0.843 | 1.037 | 1.285 | 1.1 | 0.666 | 1.017 | 0.822 | 0.858 | 0.813 | 0.831 |
| TRA(F)+TRA(N) | 1.312 | 1.16 | 0.749 | 1.074 | 1.711 | 1.303 | 0.761 | 1.258 | 0.613 | 0.785 | 0.603 | 0.667 |
| 2TRA(N) | 0.02 | 0.018 | 0.023 | 0.02 | 0.06 | 0.096 | 0.027 | 0.061 | 0.014 | 0.012 | 0 | 0.009 |
| TRB(F)+TRA(F) | 47.776 | 47.264 | 48.595 | 47.878 | 40.89 | 48.547 | 48.71 | 46.049 | 50.523 | 51.208 | 47.627 | 49.786 |
| TRB(F)+TRA(N) | 4.033 | 4.148 | 3.864 | 4.015 | 4.732 | 4.112 | 3.504 | 4.116 | 4.849 | 3.758 | 3.96 | 4.189 |
| TRB(N)+TRA(F) | 1.095 | 1.005 | 0.89 | 0.997 | 1.072 | 0.972 | 1.168 | 1.071 | 0.892 | 0.906 | 1.338 | 1.045 |
| TRB(N)+TRA(N) | 0.03 | 0.101 | 0.07 | 0.067 | 0.126 | 0.128 | 0.068 | 0.107 | 0.014 | 0.048 | 0.079 | 0.047 |
| 3TRB(F) | 0 | 0 | 0.023 | 0.008 | 0.007 | 0.011 | 0.027 | 0.015 | 0.028 | 0.024 | 0.079 | 0.044 |
| 2TRB(F)+TRB(N) | 0.01 | 0.009 | 0.047 | 0.022 | 0.02 | 0 | 0 | 0.007 | 0.028 | 0.012 | 0 | 0.013 |
| TRB(F)+2TRB(N) | 0 | 0 | 0.023 | 0.008 | 0.007 | 0 | 0.014 | 0.007 | 0 | 0 | 0 | 0 |
| 3TRB(N) | 0 | 0 | 0 | 0 | 0 | 0 | 0 | 0 | 0 | 0 | 0 | 0 |
| 3TRA(F) | 0 | 0.018 | 0 | 0.006 | 0.027 | 0 | 0 | 0.009 | 0.028 | 0 | 0.026 | 0.018 |
| 2TRA(F)+TRA(N) | 0.02 | 0.009 | 0 | 0.01 | 0.02 | 0 | 0 | 0.007 | 0.014 | 0 | 0 | 0.005 |
| TRA(F)+2TRA(N) | 0 | 0 | 0 | 0 | 0 | 0 | 0 | 0 | 0 | 0 | 0 | 0 |
| 3TRA(N) | 0 | 0 | 0 | 0 | 0 | 0 | 0 | 0 | 0 | 0 | 0 | 0 |
| Others | 27.68 | 26.049 | 24.591 | 26.107 | 22.57 | 26.991 | 25.088 | 24.883 | 20.927 | 22.596 | 20.404 | 21.309 |
| Total | 100 | 100 | 100 | 100 | 100 | 100 | 100 | 100 | 100 | 100 | 100 | 100 |
|  |  |  |  |  |  |  |  |  |  |  |  |  |
| Single TCR | 59.313 | 57.744 | 49.409 | 55.489 | 49.409 | 60.457 | 59.686 | 56.517 | 58.479 | 59.799 | 54.76 | 57.679 |
| Multiple TCR | 15.186 | 14.529 | 12.739 | 14.151 | 12.739 | 14.024 | 13.433 | 13.399 | 12.067 | 13.399 | 12.352 | 12.606 |
| Abnormal TCR | 25.502 | 27.731 | 37.851 | 30.361 | 37.851 | 25.519 | 26.882 | 30.084 | 29.454 | 26.801 | 32.887 | 29.714 |
| Total | 100 | 100 | 100 | 100 | 100 | 100 | 100 | 100 | 100 | 100 | 100 | 100 |
|  |  |  |  |  |  |  |  |  |  |  |  |  |
| One type of chain | 16.339 | 18.246 | 19.063 | 17.883 | 26.332 | 16.118 | 18.936 | 20.462 | 20.44 | 18.957 | 23.446 | 20.948 |
| Two types of chains | 55.952 | 55.669 | 56.252 | 55.958 | 51.014 | 56.877 | 55.937 | 54.609 | 58.536 | 58.408 | 56.046 | 57.663 |
| Three or more types of chains | 27.71 | 26.085 | 24.684 | 26.16 | 22.651 | 27.002 | 25.129 | 24.927 | 21.025 | 22.632 | 20.509 | 21.389 |
| Total | 100 | 100 | 100 | 100 | 100 | 100 | 100 | 100 | 100 | 100 | 100 | 100 |

Single TCR includes the following cases: TRB(F)+TRA(F)+xTRB(N)+yTRA(N); Multiple TCR includes the following cases TRB(F)+TRA(F)+TRB/TRA(F)

+x1TRB(F)+x2TRB(N)+y1TRA(F)+y2TRA(N);Abnormal TCR includes the following cases xTRB(F)、xTRB(N)、yTRA(F)、yTRA(N)、xTRB(N)+yTRA(N)、

xTRB(F)+yTRA(N)、xTRB(N)+yTRA(F)(x,x1,x2,y,y1,y2 as an integer). F: functional; N: non-functional.

| Donors | | Two antibody combinations | | | | | | | | | | | | | |
| --- | --- | --- | --- | --- | --- | --- | --- | --- | --- | --- | --- | --- | --- | --- | --- |
|  |  | CD3^+^  Vα24^+^ | CD3^+^  Vα7.2^+^ | Vα24^+^  Vα7.2^+^ | CD3^+^  Vβ13.1^+^ | CD3^+^  Vβ8^+^ | Vβ13.1^+^  Vβ8^+^ | CD3^+^  Vα3.2^+^ | CD3^+^  Vα11.1/11.2^+^ | CD3^+^  Vα2^+^ | Vα3.2^+^  Vα2^+^ | Vα2^+^  Vα11.1/11.2^+^ | CD3^+^  Vβ13^+^ | CD3^+^  Vβ5.1/5.2^+^ | Vβ13^+^  Vβ5.1/5.2^+^ |
| Human | YH1 | 3.3 | 3.6 | 0.5 | 2 | 2.8 | 0 |  |  |  |  |  |  |  |  |
|  | YH2 | 0.4 | 6.2 | 0.1 | 3 | 3 | 0 |  |  |  |  |  |  |  |  |
|  | YH3 | 0.3 | 4.4 | 0 | 3.5 | 3.2 | 0 |  |  |  |  |  |  |  |  |
|  | OH1 | 0.2 | 2.5 | 0 | 1.7 | 2.2 | 0 |  |  |  |  |  |  |  |  |
|  | OH2 | - | - | - | 2 | 4.9 | 0 |  |  |  |  |  |  |  |  |
|  | OH3 | - | - | - | 1.3 | 2.8 | 0 |  |  |  |  |  |  |  |  |
| Mouse | M1 |  |  |  |  |  |  | 0.0 | - | 3.2 | 0.0 | - | 0.7 | 2.3 | 0.0 |
|  | M2 |  |  |  |  |  |  | 0.0 | - | 2.1 | 0.0 | - | 0.5 | 2.4 | 0.0 |
|  | M3 |  |  |  |  |  |  | 0.0 | - | 1.8 | 0.0 | - | 0.3 | 1.0 | 0.0 |
|  | M4 |  |  |  |  |  |  | - | - | - | - | - | 0.5 | 1.0 | 0.0 |
|  | M5 |  |  |  |  |  |  | - | 0.0 | 2.9 | - | 0.0 | 0.5 | 2.4 | 0.0 |
|  | M6 |  |  |  |  |  |  | - | 0.0 | 3.1 | - | 0.0 | 0.2 | 1.7 | 0.0 |
|  | M7 |  |  |  |  |  |  | - | 0.0 | 3.9 | - | 0.0 | 0.4 | 2.5 | 0.0 |
|  | M8 |  |  |  |  |  |  | - | 0.0 | 3.1 | - | 0.0 | 0.2 | 2.7 | 0.0 |
|  | M9 |  |  |  |  |  |  | - | 0.0 | 3.4 | - | 0.0 | 0.4 | 2.7 | 0.0 |
|  | M10 |  |  |  |  |  |  | - | 0.0 | 4.1 | - | 0.0 | - | - | - |
|  | M11 |  |  |  |  |  |  | - | 0.0 | 3.0 | - | 0.0 | 0.3 | 2.0 | 0.0 |

Table S4 Frequency of dual β or α chains in humans and mice

Table S5 Sample information for Flow Cytometry detection of human and mouse dual TCR

| Species | Donors | Sample | Gender | Age |
| --- | --- | --- | --- | --- |
| Volunteer | YH1 | Peripheral blood | Female | 24 years |
|  | YH2 | Peripheral blood | Male | 23 years |
|  | YH3 | Peripheral blood | Female | 23 years |
|  | OH1 | Peripheral blood | Male | 72 years |
|  | OH2 | Peripheral blood | Female | 74 years |
|  | OH3 | Peripheral blood | Male | 65 years |
| Mouse | C57BL/6 1(M1) | Spleen | - | 3 months |
|  | C57BL/6 2(M2) | Spleen | - | 17 months |
|  | C57BL/6 3(M3) | Thymus | - | 3 months |
|  | C57BL/6 4(M4) | Lymph Node | - | 20 months |
|  | C57BL/6 5(M5) | Spleen | - | 18 months |
|  | C57BL/6 6(M6) | Spleen | - | 18 months |
|  | C57BL/6 7(M7) | Spleen | - | 18 months |
|  | C57BL/6 8(M8) | Spleen | - | 18 months |
|  | C57BL/6 9(M9) | Spleen | - | 18 months |
|  | C57BL/6 10(M10) | Spleen | - | 18 months |
|  | C57BL/6 11(M11) | Thymus | - | 24months |


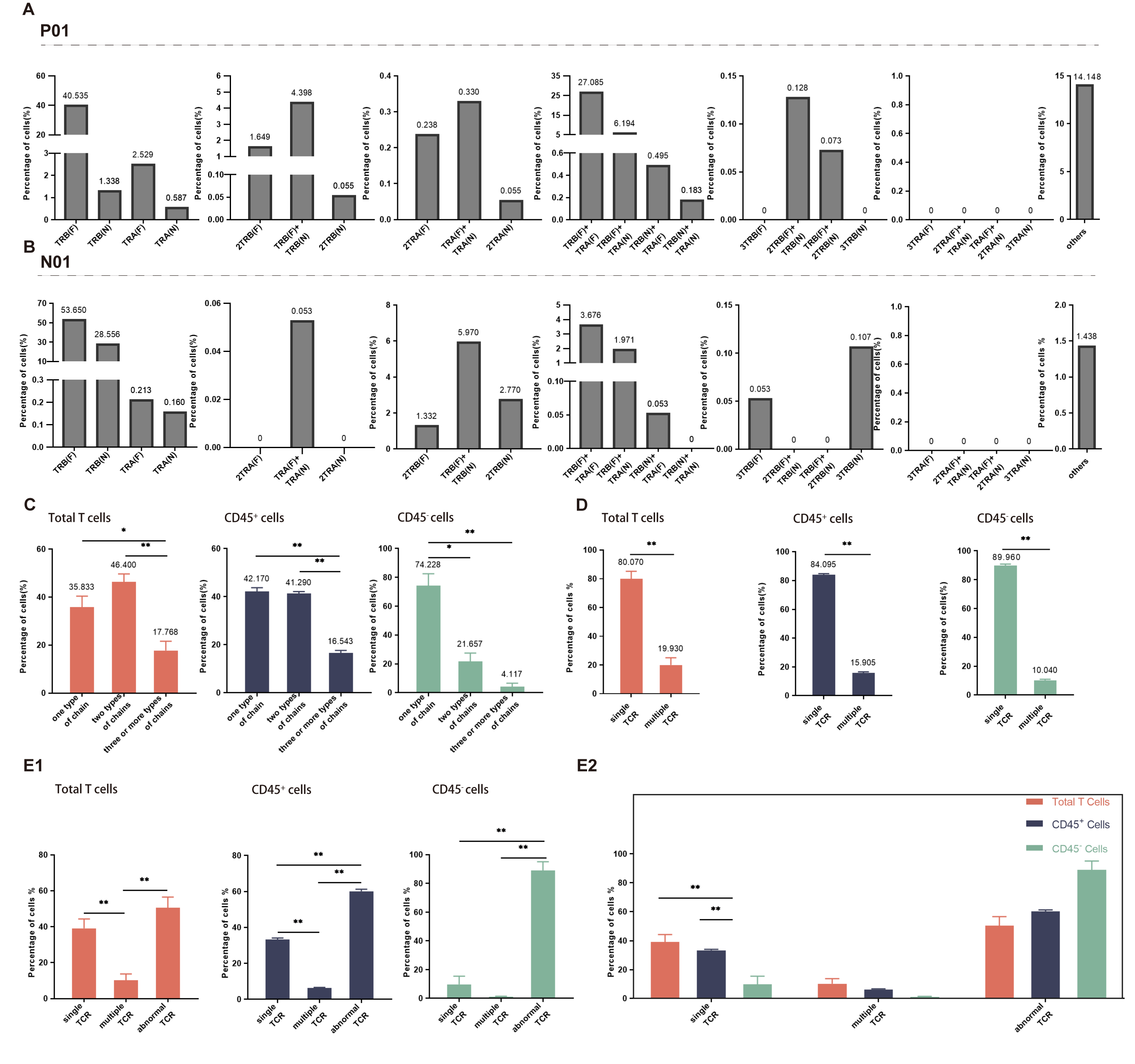


Fig. S1 Expression of TCR chains in single T cells of human thymus. A: The proportion of 23 T cells according TCR mRNA types in P01 sample; B: The proportion of 23 T cell according TCR mRNA types in N01 sample; C: The proportions of one, two, and three (or more) types of chains expression in the subsets of total T cells, CD45^+^cell, and CD45^-^ cell in humans thymus; D: The percentage of T cells with single or multiple TCR in thymus total T cells, CD45^+^ and CD45^-^ cells; E1: The proportion of T cells with single, multiple and abnormal TCR in three groups; E2: The differences in the proportion of single, multiple, and abnormal TCR T cells among the three groups. F: functional; N: non-functional.


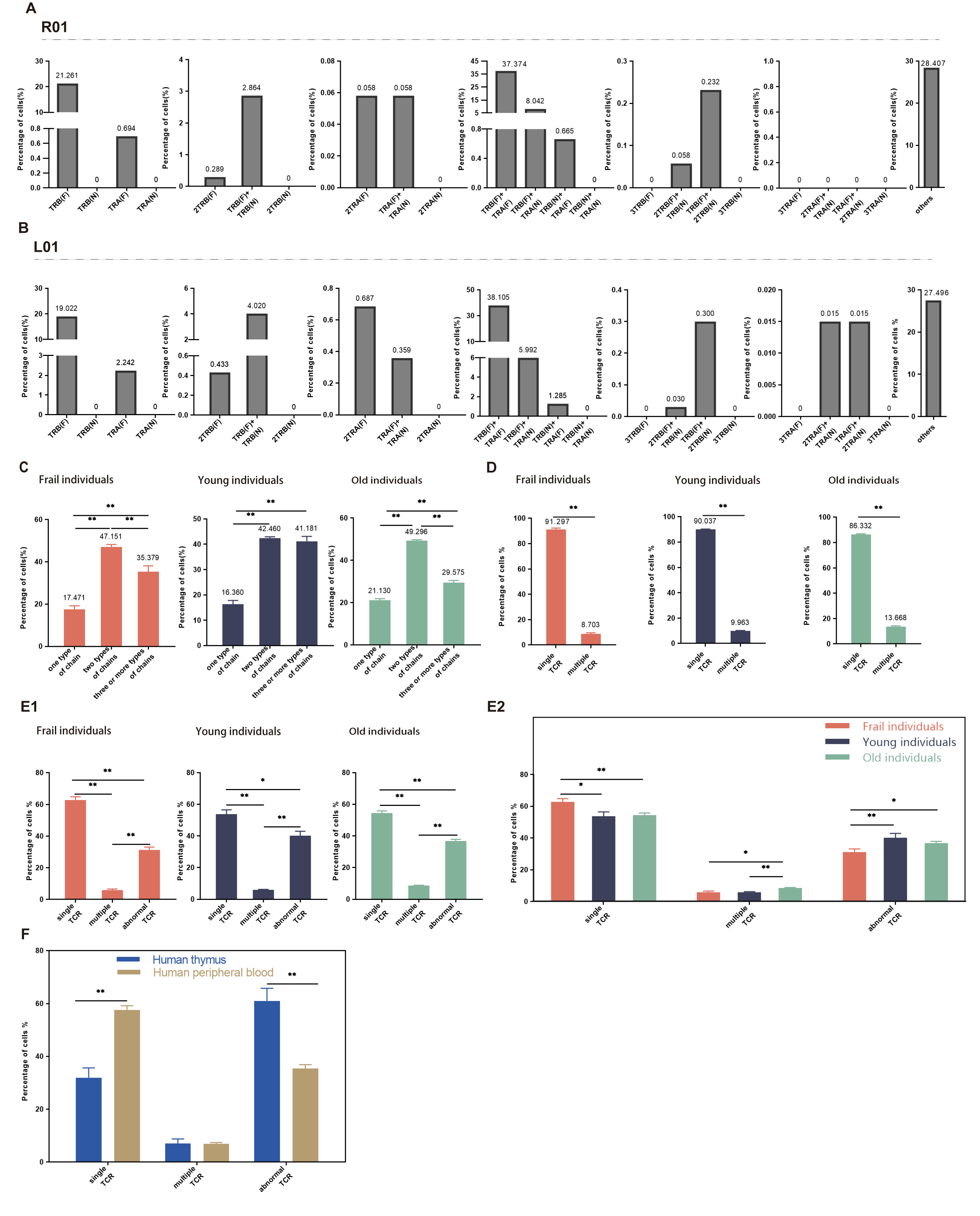


Fig. S2 Expression of TCR chains in single T cells of human peripheral blood. A: The proportion of 23 T cells according TCR mRNA types in R01 sample; B: The proportion of 23 T cell according TCR mRNA types in L01 sample; C: The proportions of one, two, and three (or more) types of chains expression in young, frail and old populations; D: The percentage of T cells with single or multiple TCR in frail, young and old populations; E1: The proportion of T cells with single, multiple and abnormal TCR in three groups; E2: The differences in the proportion of single, multiple, and abnormal TCR T cells among the three groups; F: The differences in the proportion of single, multiple, and abnormal TCR T cells between human thymus and human peripheral blood. F: functional; N: non-functional.


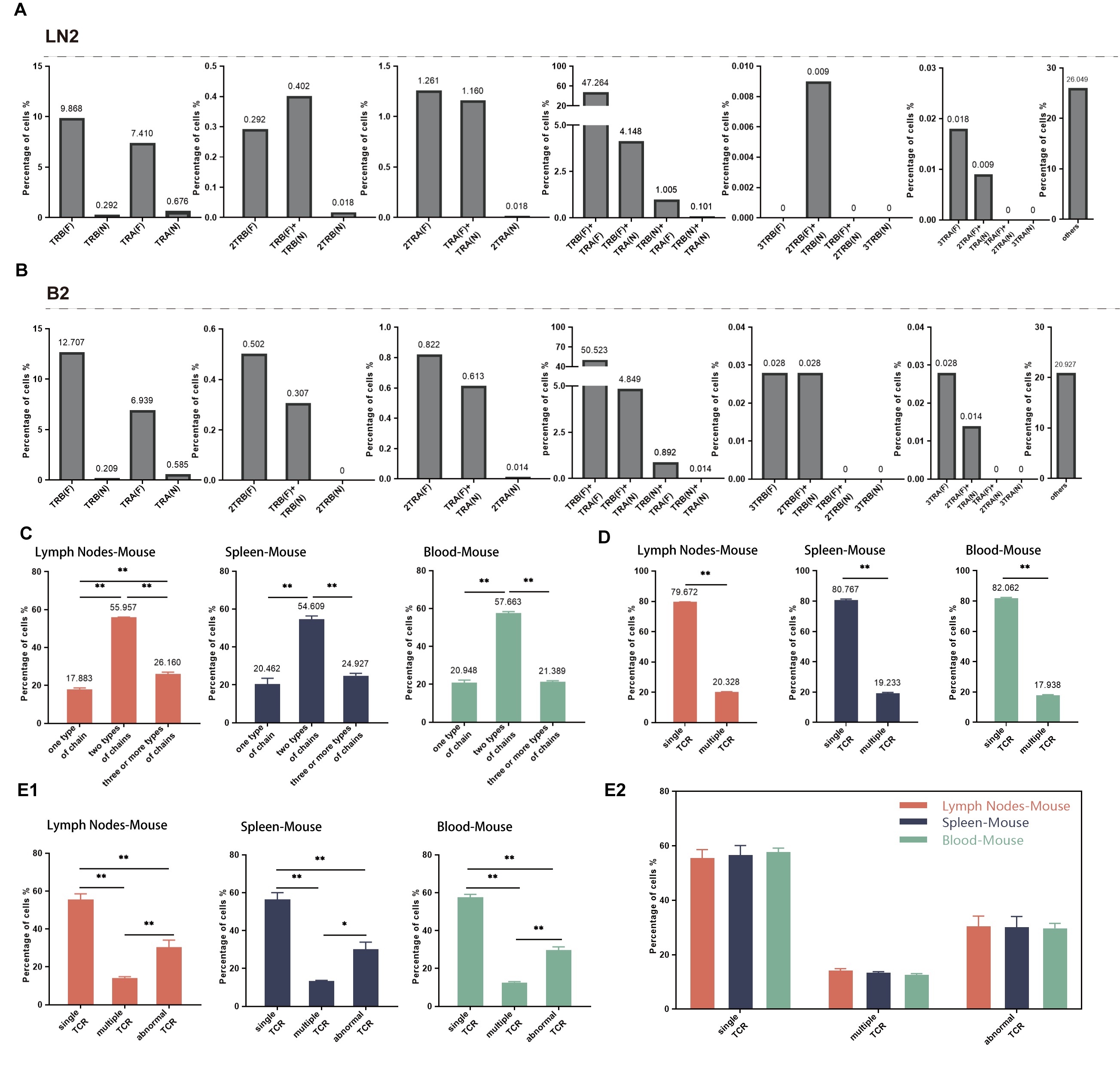


Fig. S3 Expression of TCR chain in single T cell of mouse lymphoid tissue. A: The proportion of 23 T cells according TCR mRNA types in LN2 sample; B: The proportion of 23 T cell according TCR mRNA types in B1 sample; C: The proportions of one, two, and three (or more) types of chains expression in lymph nodes, spleen and peripheral blood; D: The percentage of T cells with single or multiple TCR in lymph nodes, spleen and peripheral blood; E1: The proportion of T cells with single, multiple and abnormal TCR in three groups; E2: The differences in the proportion of single, multiple, and abnormal TCR T cells among the three groups. F: functional; N: non-functional.


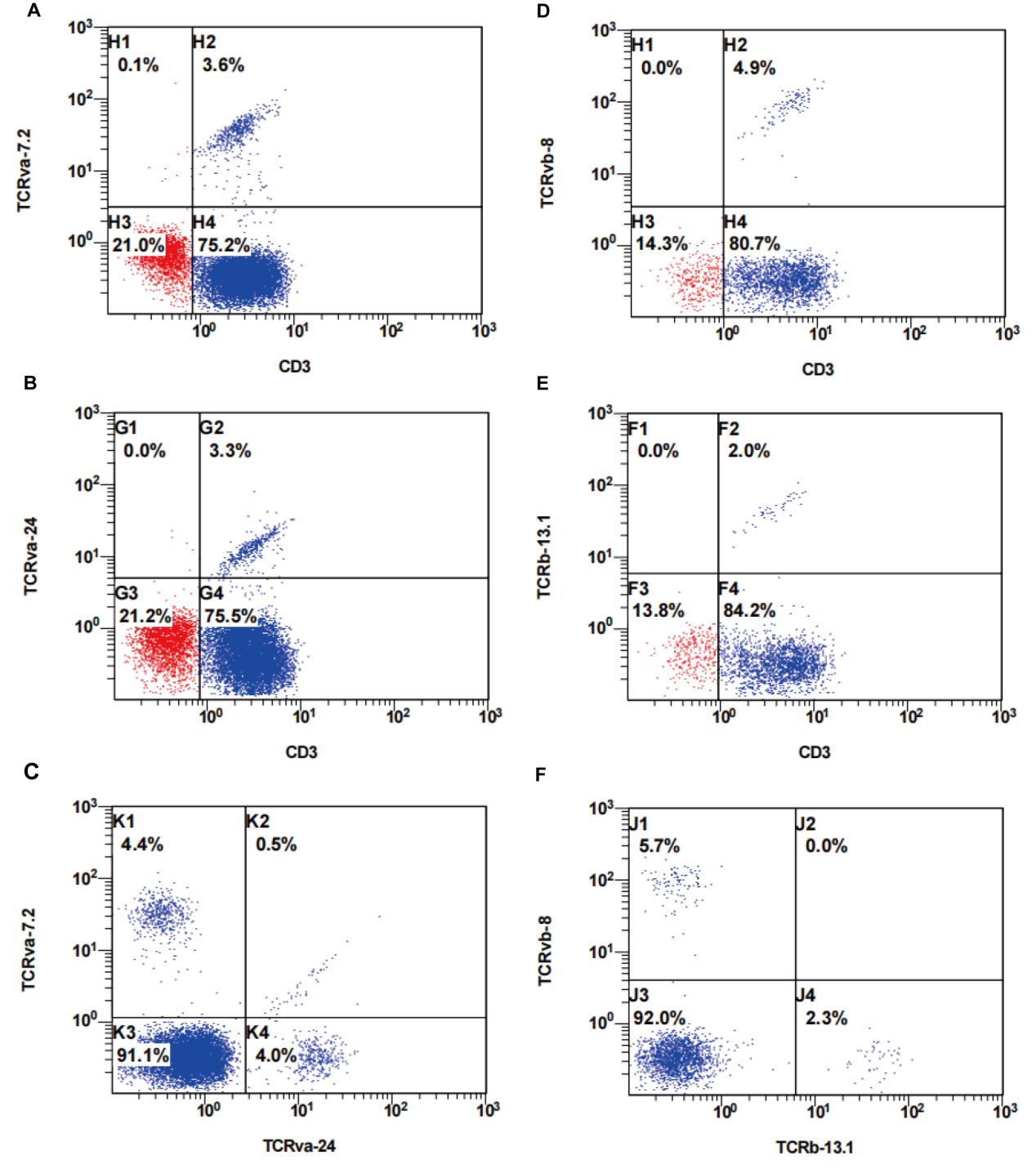


Fig. S4 The flow cytometry detection results of dual TCR chains in human peripheral blood. A-C: Cells were stained with APC-TCRVα7.2, PC5.5-TCRVα24, FITC-CD3; D-F: PE-TCRVβ13.1 and APC-TCRVβ8 were analyzed by FACS. Shown are representative dot plots of log fluorescence data for stained peripheral blood cells gated on lymphocytes on the basis of forward and side scatter. 0.5% TCRVα7.2^+^ and TCRVα24^+^ T cells were observed, however, no TCRβ13.1^+^ TCRVβ8^+^ T cells were detected.


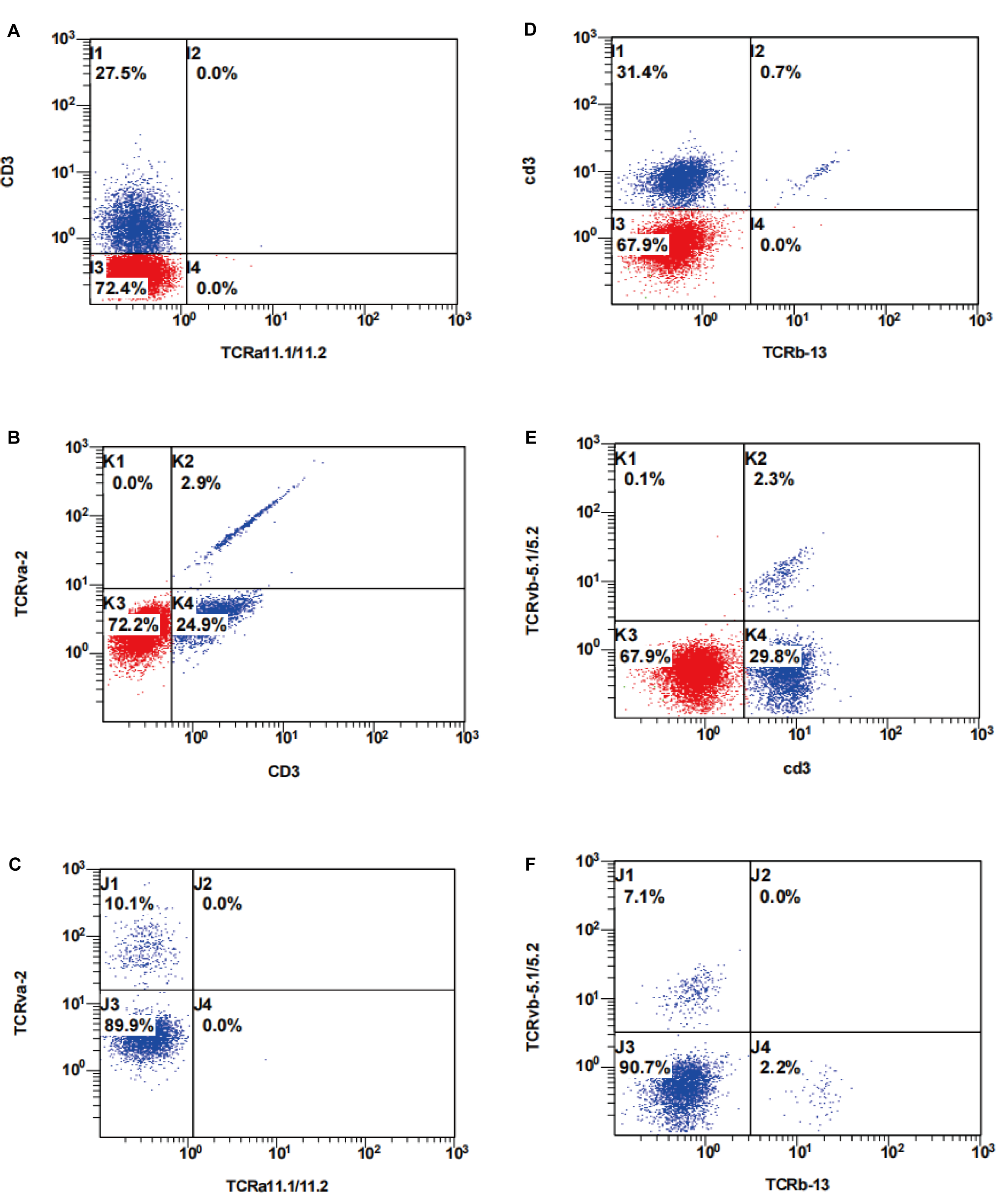


Fig. S5 The flow cytometry detection results of dual TCR chains in mouse spleen. A-C: Cells were stained with PE-TCRVα11.1/11.2, APC-TCRVα2, PC5.5-CD3; D-F: PE-TCRVβ13 and APC-TCRβ5.1/5.2 were analyzed by FACS. Shown are representative dot plots of log fluorescence data for stained spleen cells gated on lymphocytes on the basis of forward and side scatter. However, we did not detect the presence of TCRVα11.1/11.2^+^ and TCRVα2^+^ T cells, as well as T cells expressing dual β chains (TCRβ5.1/5.2^+^TCRVβ13^+^).
